# A novel mechanism of generating extracellular vesicles during apoptosis via a beads-on-a-string membrane structure

**DOI:** 10.1038/ncomms8439

**Published:** 2015-06-15

**Authors:** Georgia K. Atkin-Smith, Rochelle Tixeira, Stephanie Paone, Suresh Mathivanan, Christine Collins, Michael Liem, Katharine J. Goodall, Kodi S. Ravichandran, Mark D. Hulett, Ivan K.H. Poon

**Affiliations:** 1 Department of Biochemistry and Genetics, La Trobe Institute for Molecular Science, La Trobe University, Melbourne, Victoria 3086, Australia; 2 Department of Microbiology, Center for Cell Clearance, University of Virginia, Charlottesville, Virginia 22908, USA

## Abstract

Disassembly of apoptotic cells into smaller fragments (a form of extracellular vesicle called apoptotic bodies) can facilitate removal of apoptotic debris and intercellular communication. However, the mechanism underpinning this process is unclear. While observing monocytes undergoing apoptosis by time-lapse microscopy, we discovered a new type of membrane protrusion that resembles a ‘beads-on-a-string' structure. Strikingly, the ‘beads' are frequently sheared off the ‘string' to form apoptotic bodies. Generation of apoptotic bodies via this mechanism can facilitate a sorting process and results in the exclusion of nuclear contents from apoptotic bodies. Mechanistically, generation of ‘beads-on-a-string' protrusion is controlled by the level of actomyosin contraction and apoptopodia formation. Furthermore, in an unbiased drug screen, we identified the ability of sertraline (an antidepressant) to block the formation of ‘beads-on-a-string' protrusions and apoptotic bodies. These data uncover a new mechanism of apoptotic body formation in monocytes and also compounds that can modulate this process.

## Abstract (web summary)

During apoptosis, cells break up into smaller fragments to facilitate removal. Here the authors characterize a beads-on-a-string structure formed by monocytes undergoing apoptosis *in vitro*, which shears into apoptotic bodies lacking nuclear contents, and is blocked by the antidepressant sertraline.

## Graphical abstract

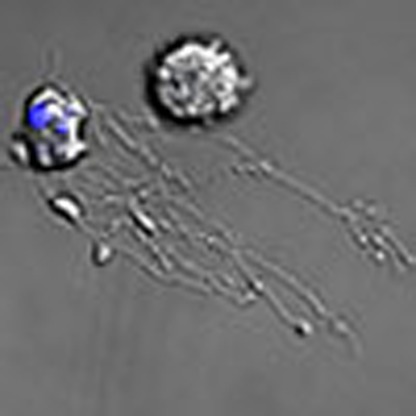

Apoptosis (programmed cell death) occurs throughout life in essentially all tissues as part of normal development, homeostasis and pathogenic processes^1^,^2^,^3^. During the later stages of apoptosis, certain cell types such as T lymphocytes can disassemble into smaller membrane-bound extracellular vesicles (∼1–5 μm in diameter) called apoptotic bodies, a step that could aid efficient clearance of corpses by phagocytes^4^,^5^. Cellular contents such as cytokines, mircoRNA and DNA can also be packaged into apoptotic bodies to regulate immunity, tissue repair and tumorigenesis^6^,^7^,^8^. Thus, the generation of apoptotic bodies by certain cell types (for example, T lymphocytes and endothelial cells) can promote apoptotic cell clearance and mediate intercellular communication. However, the precise mechanism underpinning the formation of apoptotic bodies is not well defined.

The generation of apoptotic bodies is generally considered as a stochastic process, involving the initial formation of membrane blebs (a circular bulge at the plasma membrane) and subsequent separation of blebs to generate apoptotic bodies^9^,^10^,^11^. Recently, we demonstrated that the formation of apoptotic bodies is a highly regulated multi-step process in T lymphocytes, involving distinct morphological and biochemical steps^12^. In particular, we discovered a new type of ‘string-like' membrane protrusion (known as apoptopodia) that forms exclusively after the onset of membrane blebbing and facilitates the separation of blebs to generate individual apoptotic bodies^12^. We also identified the caspase-activated pannexin 1 (PANX1) channel^13^ as a key negative regulator of apoptopodia formation in T cells and thymocytes^12^. Blockade of PANX1 channel activity during apoptosis through genetic and pharmacological approaches promotes the formation of apoptopodia and apoptotic bodies^12^.

Here we describe a new mechanism of generating apoptotic bodies by monocytes via the formation of a novel ‘beads-on-a-string' membrane structure (preliminarily denoted as ‘beaded apoptopodia'). Cell biologic and proteomic analysis revealed that the generation of apoptotic bodies via this mechanism facilitates a sorting process to regulate the localization of certain intracellular contents into apoptotic bodies. Furthermore, we describe a new paradigm that a fine control of two key steps in apoptotic cell disassembly (that is, membrane blebbing^9^,^14^,^15^ and apoptopodia formation^12^) regulates the formation of beaded apoptopodia. In addition, in an unbiased screen for pharmacological compounds that can inhibit the apoptotic cell disassembly process, we identify the ability of a commonly prescribed antidepressant sertraline to block the formation of beaded apoptopodia and apoptotic bodies without affecting other hallmarks of apoptosis, possibly by interfering with intracellular vesicle transport.

## Results

### Formation of novel protrusions by apoptotic monocytes

**Figure 1:**
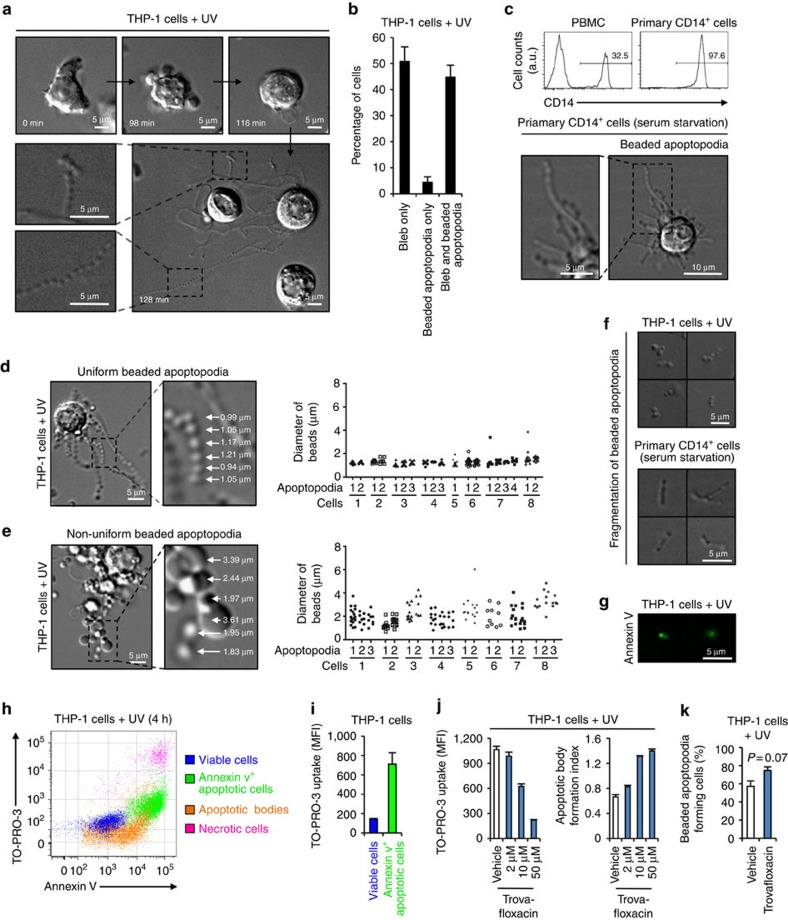
Formation of beaded apoptopodia by apoptotic human monocytes. (**a**) Time-lapse images monitoring THP-1 monocytes undergoing ultraviolet-induced apoptosis. (**b**) Quantitation of live microscopy data to determine the percentage of apoptotic THP-1 cells that form membrane bleb and/or beaded apoptopodia during 4 h of time-lapse imaging (*n*=3). (**c**) Upper, isolation of primary human CD14^+^ cells from peripheral blood mononuclear cells. Lower, primary CD14^+^ cells undergoing spontaneous apoptosis during serum starvation. DIC images of apoptotic THP-1 cells forming uniform beaded apoptopodia (**d**) or non-uniform beaded apoptopodia (**e**) 2 h post ultraviolet irradiation. Quantitation of the diameter of vesicle-like structures on each type of beaded apoptopodia is shown on the right. (**f**) Fragmentation of beaded apoptopodia from apoptotic THP-1 and primary CD14^+^ cells under cultured conditions. (**g**) Images of apoptotic bodies stained with annexin V (green). (**h**) Flow cytometry analysis showing each type of cells and apoptotic particles gated according to Supplementary Fig. 3. (**i**) TO-PRO-3 dye uptake in viable and annexin V^+^ apoptotic THP-1 cells (*n*=3). (**j**) TO-PRO-3 uptake and formation of apoptotic bodies from apoptotic THP-1 cells treated with the PANX1 inhibitor trovafloxacin (*n*=3). (**k**) Quantitation of the percentage of untreated or trovafloxacin-treated apoptotic THP-1 cells that forms beaded apoptopodia (*n*=3). Error bars represent s.e.m. Data are representative of at least two independent experiments. *P*=0.07, unpaired Student's two-tailed *t*-test.

Previous studies have shown that the disassembly of T lymphocytes into apoptotic bodies is a highly organized process induced by simultaneous formation of both membrane blebbing and apoptopodia^12^,^16^. We asked whether other leukocytes, in particular monocytes, can also generate apoptotic bodies through a similar mechanism. We first sought to monitor human monocytic-like THP-1 cells undergoing apoptosis by time-lapse differential interference contrast (DIC) microscopy. THP-1 cells initially displayed membrane-blebbing morphology at ∼90 min after induction of apoptosis by ultraviolet irradiation (Fig. 1a and Supplementary Fig. 1a). Unlike the drastic change in morphology of apoptotic T cells undergoing membrane blebbing^9^,^12^, only small membrane blebs appeared on the surface of apoptotic THP-1 cells (Fig. 1a and Supplementary Fig. 1a). Unexpectedly, at a later stage of apoptosis when membrane blebbing was beginning to cease, multiple thin and narrow membrane structures protruded rapidly and simultaneously from the apoptotic THP-1 cells (Fig. 1a and Supplementary Fig. 1a). Strikingly, these membrane protrusions extended up to eight times the length of the apoptotic cell and adopted a remarkable ‘beads-on-a-string' morphology (Fig. 1a and Supplementary Fig. 1b). As these ‘beads-on-a-string' membrane protrusions bare resemblance to the recently described apoptopodia on apoptotic T lymphocytes^12^, we preliminarily denote them as ‘beaded apoptopodia'. It is worth noting that the formation of beaded apoptopodia is not a rare event but occurred in ∼45% of cells that had undergone membrane blebbing (Fig. 1b). Furthermore, formation of beaded apoptopodia is not exclusive to monocytic cells induced to undergo apoptosis by ultraviolet treatment and were observed on THP-1 cells and primary human CD14^+^ monocytes undergoing spontaneous apoptosis under serum-free conditions (Fig. 1c and Supplementary Fig. 1c). It is worth noting that beaded apoptopodia were not found in other cell types including neuronal cells, squamous epithelial cells and cervical epithelial cells during the progression of apoptosis (Supplementary Fig. 2).

Beaded apoptopodia generated from apoptotic monocytes can be further categorized into two subclasses, uniform or non-uniform beaded apoptopodia, based on whether the ‘beads' or vesicle-like structures on the apoptopodia are of the same or different sizes, respectively (Fig. 1d,e). Interestingly, ‘beads' found on uniform beaded apoptopodia are predominately 1 μm in diameter, with the exception of the larger ‘bead' at the tip of each strand of apoptopodia (Fig. 1d). It is worth noting that the diameter of ‘beads' was similar between apoptopodia strands on the same cells and between apoptopodia on different apoptotic monocytes (Fig. 1d). In contrast, ‘beads' found on non-uniform beaded apoptopodia exhibited no obvious pattern in size, with diameters ranging between 1 and 4 μm (Fig. 1e). Importantly, after the formation of both uniform and non-uniform beaded apoptopodia on apoptotic monocytes, the ‘beads' could often fragment and consequently release sections of the apoptopodia or individual vesicles (Fig. 1f and Supplementary Fig. 1a). As these vesicles were generated from apoptotic cells (Fig. 1a and Supplementary Fig. 1a,d–f), positive for annexin V staining (indicative of phosphatidylserine exposure) (Fig. 1g) and ∼1–4 μm in diameter (Fig. 1f,g and Supplementary Fig. 1a), they were classified as apoptotic bodies. Thus, these observations suggest that apoptotic bodies can be generated through the fragmentation of a ‘beads-on-a-string' membrane protrusion.

We next monitored the generation of apoptotic bodies from monocytic cells using a recently described flow cytometry approach^12^ (Fig. 1h and Supplementary Fig. 3). Consistent with microscopic analysis (Fig. 1g), apoptotic bodies were found to expose intermediate level of phosphatidylserine compared with apoptotic cells (Fig. 1h). Apoptotic cells also showed an approximately five fold increase (relative to viable cells) in TO-PRO-3 dye uptake (Fig. 1i), an indirect measurement of the activation of caspase-activated PANX1 channels during apoptosis^12^,^13^. We demonstrated previously that PANX1 is a key negative regulator of apoptotic body formation in T cells and blockade of PANX1 can promote apoptotic cell disassembly and apoptopodia formation^12^. Similarly, inhibition of PANX1 by the quinolone antibiotic trovafloxacin^12^ markedly reduced TO-PRO-3 dye uptake in apoptotic monocytes and enhanced the formation of apoptotic bodies (Fig. 1j). Even though monocytes can generate apoptopodia under basal condition during apoptosis (Fig. 1a-e and Supplementary Fig. 1a-c), an increasing trend in apoptopodia formation was also observed when PANX1 channels were blocked (Fig. 1k). It is worth noting that blockade of PANX1 channels by trovafloxacin does not affect the level of THP-1 cell apoptosis (Supplementary Fig. 4a,b), ruling this out as a cause of increased apoptotic body formation.

### Nuclear material is excluded from beaded apoptopodia

**Figure 2:**
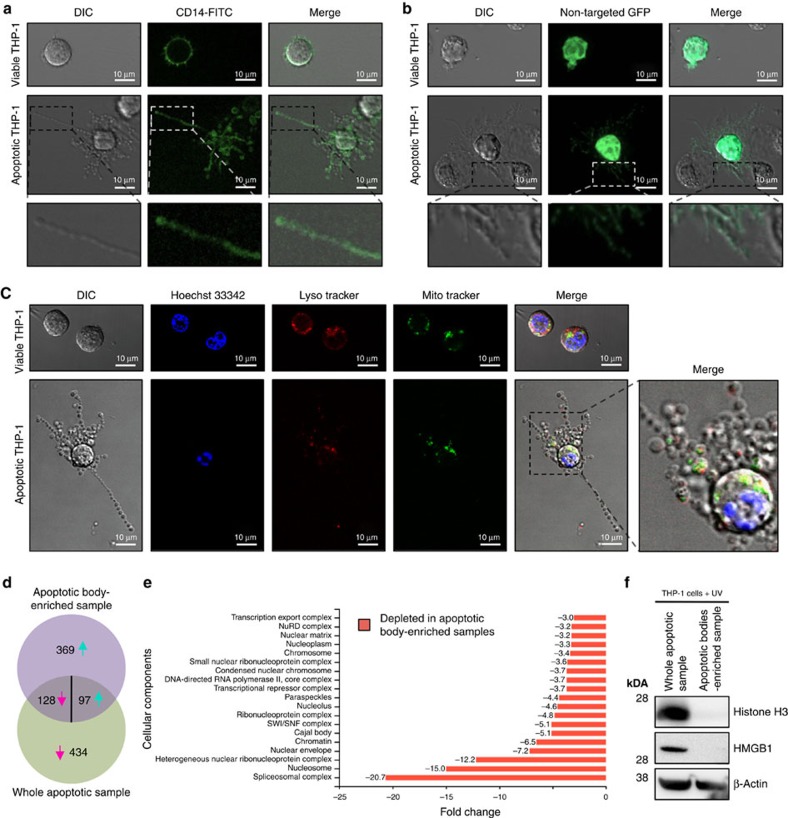
Exclusion of nuclear contents in beaded apoptopodia. Localization of surface CD14 (**a**) and non-targeted intracellular GFP (**b**) in viable and apoptotic THP-1 monocytic cells. (**c**) Localization of Hoechst 33342, LysoTracker Red and MitoTracker Green staining in viable and apoptotic THP-1 cells. (**d**) Venn diagram of differentially abundant proteins in whole apoptotic sample and apoptotic body-enriched sample (a list of proteins identified in the proteomic analysis is shown in Supplementary Data 1). A total of 562 proteins were of low abundance in apoptotic body-enriched sample compared with whole apoptotic sample. Magenta and turquoise arrows indicate proteins that are in low or high abundance in apoptotic body-enriched sample compared with whole apoptotic sample, respectively. (**e**) Subcellular components depleted in apoptotic body-enriched sample compared with whole apoptotic sample are depicted based on FunRich analysis software. **(f)** Levels of histone H3, HMGB1 and β-actin in whole apoptotic sample and apoptotic body-enriched sample. (**a–c**,**f**) Data are representative of at least three independent experiments.

Packaging of intracellular organelles such as the mitochondria and nucleus into apoptotic bodies is generally thought to be a stochastic process^10^,^11^. Nevertheless, distribution of nuclear contents into apoptotic bodies has been suggested to promote pathological conditions including autoimmunity^17^,^18^ and tumorigenesis^8^. Therefore, we asked which subcellular compartments can be localized to the beaded apoptopodia. Initially, we monitored the distribution of the plasma membrane and general intracellular contents in apoptotic monocytes by staining the cell surface protein CD14 and expressing the non-targeted green fluorescent protein (GFP), respectively. CD14 was detected on the surface of apoptotic cell body (that is, the larger part of the apoptotic cell where beaded apoptopodia is protruding from) and beaded apoptopodia (Fig. 2a). Non-targeted GFP (localized to both the cytoplasm and the nucleus) was also found in the apoptotic cell body and throughout the entire beaded apoptopodia (Fig. 2b). Localization of nuclear DNA, mitochondria and acidic organelles were monitored in apoptotic cells by Hoechst 33342, MitoTracker and LysoTracker staining, respectively. Nuclear DNA was found exclusively in the apoptotic cell body and not in beaded apoptopodia (Fig. 2c and Supplementary Fig. 5), whereas mitochondria and acidic organelles could be observed in the cell body and sporadically in beaded apoptopodia (Fig. 2c). Distribution of nuclear contents and mitochondria in apoptotic monocytes were also confirmed by tracking the localization of GFP-histone H2B, GFP-histone H4 and Mito-DsRed (Supplementary Fig. 6).

As apoptotic bodies generated from apoptotic monocytes appear to originate solely from beaded apoptopodia (Fig. 1a,f,g and Supplementary Fig. 1a), proteomic analysis of purified apoptotic bodies could help determine the protein composition of beaded apoptopodia. To address this, we derived a method to enrich apoptotic bodies by differential centrifugation (Supplementary Fig. 7) and liquid chromatography–tandem mass spectrometry (LC-MS/MS)-based label-free quantitative proteomic analysis was performed on apoptotic body-enriched sample compared with whole apoptotic sample. A total of 1,028 proteins were differentially abundant in the samples (Supplementary Data 1; data deposited in Vesiclepedia^19^), with 562 and 466 proteins found in high abundance in whole apoptotic sample and apoptotic body-enriched sample, respectively (Fig. 2d). Consistent with cell biological analysis (Fig. 2c and Supplementary Figs 5 and 6a,b), nuclear components were markedly depleted in apoptotic body-enriched sample (Fig. 2e and Supplementary Fig. 8a). Among the proteins that are of high abundance, 51.6% and 5.9% were implicated in ‘regulation of nucleobase, nucleoside, nucleotide and nucleic acid metabolism' in whole apoptotic and apoptotic body-enriched samples, respectively (Supplementary Fig. 8b). Depletion of nuclear components in apoptotic body-enriched sample was further confirmed by immunoblotting for nuclear proteins such as histone H3 and HMGB1 (Fig. 2f). On the contrary, the apoptotic bodies were enriched with proteins implicated in signal transduction, cell growth and maintenance, and transport (Supplementary Fig. 8b). In addition, SH2, SH3, kinase, RhoGEF, TPR and PH domain-containing proteins were enriched in apoptotic bodies (Supplementary Fig. 8c). Collectively, these data suggest that sorting cellular components into apoptotic bodies is not a stochastic process for apoptotic monocytes and certain organelle(s), in particular the nucleus, can be excluded from apoptotic bodies.

### Blebbing and PANX1 controls beaded apoptopodia formation

**Figure 3:**
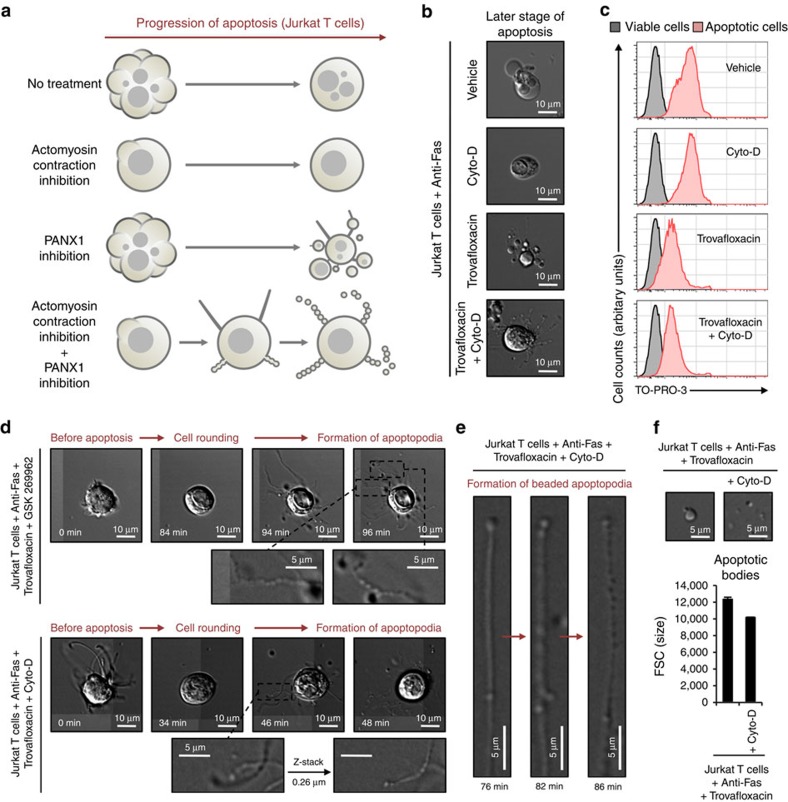
Formation of beaded apoptopodia is regulated by the balance of actomyosin contraction and PANX1 activity. (**a**) Schematic of Jurkat cells (T cell line) undergoing apoptosis under conditions when actomyosin contraction and/or PANX1 functions are impaired based on time-lapse microscopy. (**b**) Images of cells at late stage of apoptosis when actomyosin contraction and/or PANX1 functions are blocked by pharmacological compounds. (**c**) Uptake of TO-PRO-3 by apoptotic cells treated with cytochalasin D (Cyto-D) and/or trovafloxacin. (**d**) Time-lapse images monitoring progression of apoptotic cell morphology when actomyosin contraction and PANX1 functions are blocked by pharmacological compounds. **(e)** Formation of beaded apoptopodia from non-beaded-apoptopodia. (**f**) Formation of smaller apoptotic bodies through fragmentation of beaded apoptopodia (*n*=3). Error bars represent s.e.m. Data are representative of at least two independent experiments.

Previous studies have shown that the generation of apoptotic bodies from T lymphocytes involves a multi-step process^12^ (shown schematically in Supplementary Fig. 9a). Apoptotic T cells first undergo dynamic blebbing (denoted as Step 1) to form large membrane blebs and subsequent apoptopodia formation (denoted as Step 2) facilitates the separation of these blebs. Finally, separation of apoptopodia-connecting blebs (denoted as Step 3) generates apoptotic bodies that are relatively large in size (∼5 μm)^12^. In contrast, apoptotic monocytes appear to generate apoptopodia in the absence of large blebs and subsequently form beaded apoptopodia that fragment into smaller apoptotic bodies (∼1–3 μm) (Fig. 1a,f and Supplementary Fig. 1a). These observations suggest an intriguing possibility that the generation of apoptopodia (Step 2) in the absence of dynamic membrane blebbing (Step 1) may promote beaded-apoptopodia formation and a mechanism to control the size of apoptotic bodies (Supplementary Fig. 9b). To test this concept, we used apoptotic Jurkat T cells as a model system, as it does not normally generate beaded apoptopodia^12^. Strikingly, when membrane blebbing (Step 1) was inhibited by drugs that block actomyosin contraction (cytochalasin D^15^ and GSK 269962 (ref. 20)) and apoptopodia formation (Step 2) was enhanced by PANX1 inhibitor trovafloxacin^12^, beaded apoptopodia were found on T cells at a later stage of apoptosis (Fig. 3a–d). Under these conditions, we also observed the transformation of a non-beaded apoptopodia to a beaded apoptopodia within 10 min (Fig. 3e). Apoptotic bodies generated from beaded apoptopodia on T cells were smaller than those that are formed from large membrane blebs (Fig. 3f). Thus, the mechanism of apoptotic body formation and the quality of apoptotic bodies is regulated by distinct morphological steps during apoptosis.

### Identification of compounds that block apoptopodia formation

**Figure 4:**
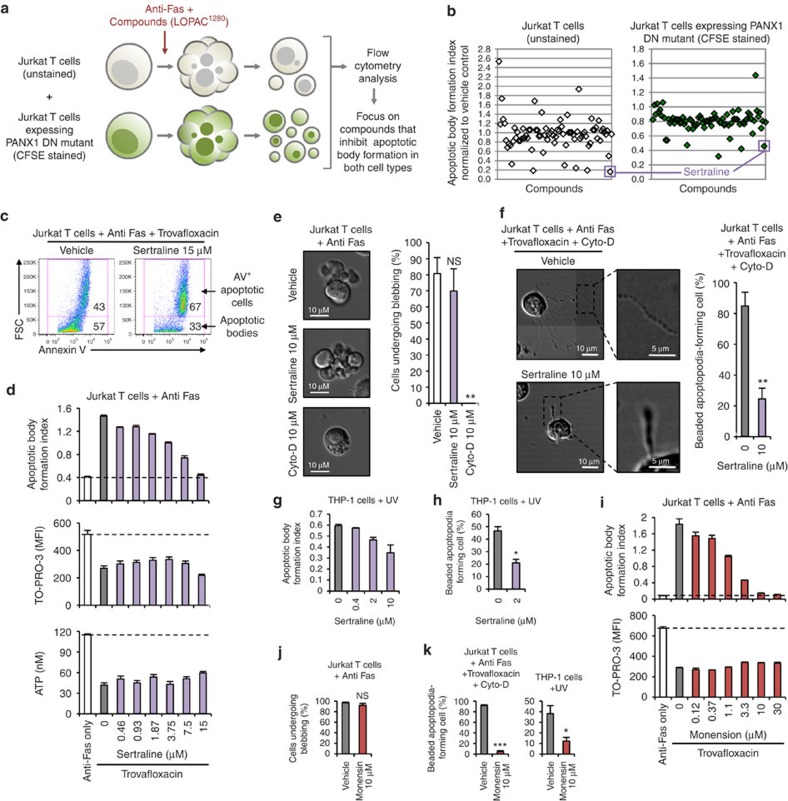
Inhibition of apoptotic cell disassembly by drugs that could interfere with vesicular transport. (**a**) Schematic representation of the drug screen approach to identify compounds that could inhibit the disassembly of apoptotic Jurkat and PANX1 DN mutant expressing Jurkat cells. (**b**) Comparison of a selected panel of compounds from the LOPAC^1280^ library in modulating the formation of apoptotic bodies from apoptotic Jurkat and PANX1 DN mutant expressing Jurkat cells. **(c)** Sertraline reduces apoptotic body formation by apoptotic Jurkat cells under conditions when PANX1 channels are blocked. (**d**) Dose-dependent inhibition of apoptotic body formation by sertraline (*n*=3). Sertraline does not interfere with TO-PRO-3 uptake by apoptotic cells or ATP release into the supernatant under conditions when PANX1 channels are blocked (*n*=3). **(e)** Left, time-lapse images monitoring membrane blebbing of apoptotic Jurkat cells treated with or without sertraline or cytochalasin D (Cyto-D). Right, percentage of apoptotic Jurkat cells (based on cell rounding morphology) that has undergone membrane blebbing during 4-h time-lapse imaging (*n*=3). **(f)** Left, representative 4-h time-lapse images monitoring beaded apoptopodia formation by Jurkat cells treated with or without sertraline. Right, percentage of apoptotic Jurkat cells forming beaded apoptopodia (*n*=3). Sertraline inhibits the formation of apoptotic bodies **(g)** and beaded apoptopodia **(h)** by apoptotic THP-1 cells (*n*=3). **(i)** Vesicle transport inhibitor monensin reduces apoptotic body formation but not TO-PRO-3 uptake by apoptotic Jurkat cells under conditions when PANX1 channels are blocked (*n*=3). (**j**) Monensin does not interfere with membrane blebbing during apoptosis progression (*n*=3). (**k**) Monensin inhibits beaded-apoptopodia formation by Jurkat and THP-1 cells (*n*=3). (**f**,**k**) Actomyosin contraction and PANX1 channels are inhibited to promote the formation of beaded-apoptopodia in Jurkat cells. Error bars represent s.e.m. (**c–k**) Data are representative of at least two independent experiments. *P<0.05, **P<0.01, ***P<0.001, NS=*P*>0.5, unpaired Student's two-tailed *t*-test.

We next wanted to perform a drug screen to identify small molecules that can inhibit apoptopodia formation and potentially elucidate the molecular machinery that underpins this key process in apoptotic cell disassembly. As monitoring the generation of apoptopodia by time-lapse microscopy is a low-throughput process, we developed a flow cytometry-based method to screen for compounds that can inhibit apoptotic body formation from apoptotic T cells (a process dependent on both membrane blebbing and apoptopodia^12^). A mixture of normal Jurkat T cells (forms low level of apoptotic bodies) and Jurkat T cells stably expressing the PANX1 dominant negative (DN) mutant (forms high level of apoptotic bodies)^12^ were induced to undergo apoptosis by anti-Fas treatment in the presence of small molecules from a Library of Pharmacologically Active Compounds (LOPAC^1280^) (Fig. 4a). We next focused on compounds that can block apoptotic body formation from both cell types, as well as those that are not known kinase inhibitors (as they may interfere with kinases such as ROCK1, PAK2 and LIMK1 that promote membrane blebbing^9^,^14^,^21^,^22^). More than 50 compounds were identified in the initial screen, to inhibit apoptotic body formation from both cell types, and five of these were validated in secondary screens. Among these five compounds, a relatively well-described drug sertraline, an antidepressant that is widely prescribed in the western countries^23^, was identified as a potent inhibitor of apoptotic cell disassembly (Fig. 4b,c). Sertraline inhibited the formation of apoptotic bodies in a dose-dependent manner under conditions where apoptotic body formation was enhanced (Fig. 4d). It is worth noting that sertraline had no obvious effect on the level of apoptosis (Supplementary Fig. 4c), DNA fragmentation (Supplementary Fig. 4d) and PANX1 channel activity (as measured by TO-PRO-3 uptake and ATP release assay^12^,^13^) (Fig. 4d and Supplementary Fig. 10). Importantly, sertraline did not affect the formation of membrane blebs during apoptosis (Fig. 4e), indicating that the blockade of apoptotic body formation by sertraline is independent of the membrane blebbing step.

To test the potential effect of sertraline on the formation of beaded apoptopodia in the T-cell system (as described in Fig. 3), apoptosis was induced under conditions where membrane blebbing is blocked and apoptopodia formation enhanced. Remarkably, sertraline significantly retarded the formation of beaded apoptopodia (Fig. 4f). Furthermore, sertraline inhibited the formation of apoptotic bodies and beaded apoptopodia from apoptotic monocytes (Fig. 4g,h), without affecting the level of apoptosis (Supplementary Fig. 4a,b). Besides lymphoid and myeloid cells, it is worth noting that sertraline also blocked the disassembly of apoptotic squamous epithelial (A431) cells (Supplementary Fig. 11a).

Sertraline functions primarily as a selective serotonin reuptake inhibitor to increase the concentration of extracellular serotonin and thereby promote serotonergic signalling^24^. Treatment of Jurkat T cells with increasing concentrations of serotonin or another selective serotonin reuptake inhibitor citalopram^24^ did not affect apoptotic cell disassembly (Supplementary Fig. 12), indicating serotonergic signalling is unlikely to be involved in regulating apoptotic body formation. Previous studies have also reported that sertraline may interfere with protein synthesis and vesicle transport in non-mammalian cells^25^,^26^. Treatment of cells undergoing apoptosis with cycloheximide to inhibit protein synthesis did not affect the formation of apoptotic bodies (data not shown). Strikingly, inhibition of vesicle transport by the drug monensin^27^,^28^ markedly inhibited the disassembly of apoptotic T cells (Fig. 4i), without affecting the level of apoptosis (Supplementary Fig. 4e), membrane blebbing (Fig. 4j) or PANX1 channel activity (Fig. 4i). Similar to sertraline, monensin also potently blocked the generation of beaded apoptopodia from apoptotic T cells and monocytes (Fig. 4k), and inhibited apoptotic body formation from squamous epithelial cells (Supplementary Fig. 11b). Collectively, these data suggest targeting the formation of apoptopodia, possibly by inhibiting vesicle trafficking, can attenuate the disassembly of apoptotic cells.

## Discussion

Although fragmentation of apoptotic cells into smaller apoptotic bodies is considered a morphologic hallmark of apoptosis^1^,^9^,^10^,^11^,^29^, it is unclear how this process is being regulated. The data presented here provide several new insights on this process during cell death, with important implications for the clearance and functions of apoptotic debris during development, homeostasis and pathogenic processes. This work uncovers a new ‘beads-on-a-string' membrane structure that can subsequently undergo fragmentation during apoptosis to generate apoptotic bodies. This represents a unique and novel mechanism in generating extracellular vesicles that is very different from the previously described mechanism underpinning the formation of apoptotic bodies^12^, microparticles^30^ and exosomes^31^,^32^,^33^. Our work also provide evidence to support a new conceptual framework that the mechanism of apoptotic cell disassembly is controlled by a multi-step process, membrane blebbing denoted as Step 1 and apoptopodia formation denoted as Step 2. The mechanism of apoptotic body formation and the quality of apoptotic bodies being generated is determined by whether Step 1 and Step 2 occur simultaneously or independently. Furthermore, we have identified a pharmacological approach to block the disassembly of apoptotic cells without affecting other hallmarks of apoptosis. As pathogens (for example, intracellular bacteria and viruses) and pathogen-derived proteins may reside inside or exposed on apoptotic bodies^34^,^35^,^36^, controlling the apoptotic cell disassembly process may have therapeutic relevance for certain infectious diseases.

## Methods

### Reagents

Trovafloxacin, 7-aminoactinomycin D, cytochalasin D, sertraline, cycloheximide, serotonin and citalopram were obtained from Sigma-Aldrich (MO). Other reagents were obtained as follows: anti-Fas (05-201, 250 ng ml^−1^, clone CH11, Millipore, MA), annexin V–fluorescein isothiocyanate (FITC; BD Biosciences, CA), TO-PRO-3 (Life Technologies, NY), carboxyfluorescein diacetate succinimidyl ester (CFSE; Life Technologies) and GSK 269962 (Tocris bioscience, UK).

### Mammalian cell culture

THP-1 human monocytic cells and Jurkat human T cells were cultured in RPMI medium. SH-SY5Y human neuronal cells, A431 human squamous epithelial cells and HeLa human cervical epithelial cells were cultured in DMEM medium. Culture media were supplemented with 10% fetal bovine serum, penicillin (50 U ml^−1^), streptomycin (50 μg ml^−1^) and MycoZap (Lonza, Switzerland). Mammalian cell lines were cultured at 37 °C in a humidified atmosphere with 5% CO_2_.

### Purification of primary human CD14^+^ monocytes

Primary human CD14^+^ monocytes were isolated from peripheral blood mononuclear cells by positive selection using CD14 MicroBeads^37^,^38^ (Miltenyi Biotec, Germany) in accordance with the manufacturer's instructions.

### Induction of apoptosis

To induce apoptosis, cells were treated with ultraviolet irradiation at 150 mJ cm^−2^ using the Stratagene UV Stratalinker 1,800 (Agilent Technologies, CA) and incubated at 37 °C in a humidified atmosphere with 5% CO_2_ for 0–4 h as indicated. In certain experiments, apoptosis was induced by culturing cells under serum-free conditions or by anti-Fas treatment (250 ng ml^−1^).

### Time-lapse DIC microscopy

Jurkat T cells, THP-1 monocytic cells and primary CD14^+^ monocytes were seeded into four-well Nunc Lab-Tek II chambered coverglass (Nunc, Denmark) immediately before induction of apoptosis and drug treatment. For monocytic cells, Nunc Lab-Tek II chambered coverglass were pre-coated with 1% poly-L-lysine^39^ (Sigma-Aldrich). SH-SY5Y neuronal cells, A431 squamous epithelial cells and HeLa cervical epithelial cells were seed into four-well Nunc Lab-Tek II chambered coverglass for 16 h, to allow cells to adhere before induction of apoptosis and drug treatment. Time-lapse DIC microscopy was performed at 37 °C in a humidified atmosphere with 5%–10% CO_2_ using the Zeiss Spinning Disk Confocal with × 63 oil-immersion objective. For the majority of the experiments, images were acquired every 2 min for 4 h. Image processing and data analysis were performed using the ZEN imaging software (Zeiss, Germany).

### Quantification of cell viability by flow cytometry

Samples were stained with annexin V-FITC, 7-aminoactinomycin D and TO-PRO-3 in annexin V binding buffer for 10 min at room temperature. Samples were incubated on ice before analysis on a BD FACSCanto flow cytometer (BD Biosciences). The resultant flow cytometry data were analysed by FlowJo software (Tree Star, OR) according to electronic gating strategy as described in Supplementary Fig. 3b.

### DNA fragmentation assay

DNA laddering during apoptosis was examined by agarose gel electrophoresis^40^. Viable and apoptotic THP-1 monocytic cells (1 × 10^6^ cells per ml) were pelleted for 5 min at 1,000*g* and resuspended in TES lysis buffer (100 mM Tris pH 8.0, 20 mM EDTA, 0.8% SDS) with RNase and incubated for 2 h at 37 °C. After incubation, cell lysates were treated with proteinase K and incubated for 16 h at 50 °C. DNA fragments were separated and visualized by agarose gel electrophoresis.

### ATP release assay

ATP levels in apoptotic cell supernatants were quantified using a luciferase/luciferin assay^13^ (CellTiter-Glo; Promega, WI) in accordance with the manufacturer's instructions.

### Monitoring the localization of organelles

THP-1 monocytic cells were stained with LysoTracker Red (Life Technologies), MitoTracker Green (Life Technologies), Hoechst 33342 (Life Technologies) and CD14-FITC (Miltenyi Biotec), according to manufacturer's instructions, before time-lapse imaging or induction of apoptosis. THP-1 cells were transfected with non-targeted GFP, Mito-DsRed (a kind gift from Dr Laura Osellame and Professor Mike Ryan), GFP-histone H2B and GFP-histone H4 (a kind gift from Dr Kylie Wagstaff and Professor David Jans) via Nucleofection 4D in accordance with the manufacturer's instructions. Confocal microscopy was performed at 37 °C in a humidified atmosphere with 5% CO_2_ using either the Zeiss Spinning Disk or the Zeiss LSM780 confocal microscope (Zeiss).

### Purification of apoptotic bodies

Viable THP-1 monocytic cells were pelleted at 25*g* for 10 min. The supernatant (containing apoptotic bodies, apoptotic and necrotic cells generated under cultured conditions) was discarded and cell pellet resuspended to 3.5 × 10^6^ cells per ml in RPMI+1% BSA to ∼4 ml. Apoptosis was subsequently induced by ultraviolet irradiation (150 mJ cm^−2^) and cells were incubated at 37 °C for 2 h. A small fraction (200 μl) of the cell suspension was collected as the whole apoptotic sample. The remaining suspension was subjected to centrifugation for 5 min at 25*g* and 3 ml of the supernatant (containing mainly apoptotic bodies) was subjected to another cycle of centrifugation for 5 min at 25*g*. The resultant supernatant (2 ml) was collected as the apoptotic body-enriched sample. Both whole apoptotic sample and apoptotic body-enriched sample were pelleted at 1,000*g* for 5 min and resuspended in 1 ml PBS. To determine cell viability by flow cytometry, 50 μl of each sample was collected. Remaining samples were pelleted at 1,000*g* for 5 min and stored at −80 °C. Schematic of the purification procedure is shown in Supplementary Fig. 7a.

### Sample preparation for mass spectrometry

Proteins in whole apoptotic sample and apoptotic body-enriched sample were first enriched by acetone precipitation. Briefly, ∼200 μg of protein samples were precipitated by 100% ice-cold acetone (−20 °C). Samples were mixed by vortexing and stored for 16 h at −20 °C. Precipitated protein samples were pelleted at 14,000*g* for 5 min at 4 °C. The supernatant was removed and the pellet was washed once in 800 μl of 100% ice-cold acetone (−20 °C). The resultant pellet was resuspended in 100 μl of TEAB buffer (50 mM) containing 8 M urea and sonicated for 5 min. Protein samples were then subjected to in-gel digestion and mass spectrometry^41^. Initially, protein samples (40 μg) were first separated by SDS–PAGE. The gel was then stained with brilliant blue Coomassie for 1 h and destained in 7.5% acetic acid and 20% ethanol until protein bands are clearly visible. Each lane was cut into 20 bands, further destained (in 1:1 ratio of 50% acetonitrile and 50 mM ammonium bicarbonate) and subjected to reduction (10 mM dithiothreitol, 45 min at 55 °C), alkylation (25 mM iodoacetamide, 30 min) and trypsinization (750 ng sequencing grade typsin, 16 h at 37 °C). Peptides were extracted from gel fragments using 25 mM ammonium bicarbonate with 0.1% trifluoroacetic acid.

### Liquid chromatography–tandem mass spectrometry

Extracted tryptic peptides from each gel band were concentrated to ∼20 μl by centrifugal lyophilization and analysed by LC-MS/MS using LTQ Orbitrap Elite mass spectrometer (Thermo Scientific, MA) fitted with nanoflow reversed-phase-HPLC (Model 1,200, Agilent). The nano-LC system was equipped with an Acclaim Pepmap nano-trap column (Dionex—C18, 100 Å, 75 μm × 2 cm) and an Acclaim Pepmap RSLC analytical column (Dionex—C18, 100 Å, 75 μm × 15 cm). Typically for each LC-MS/MS experiment, 1 μl of the peptide mix was loaded onto the enrichment (trap) column at an isocratic flow of 3 μl min^−1^ of 3% acetonitrile containing 0.1% formic acid for 4 min before the enrichment column is switched in-line with the analytical column. The eluents used for the LC were 0.1% v/v formic acid (solvent A) and 100% acetonitrile/0.1% formic acid v/v. The gradient used was 3% B to 8% B for 1 min, 8% B to 35% B in 30 min, 35% B to 85% B in 5 min and maintained at 85% B for the final 5 min. All spectra were acquired in positive mode with full-scan MS spectra scanning from *m/z* 300–2,000 in the Fourier transform (FT) mode at 30,000 resolution after accumulating to a target value of 1.00e6 with maximum accumulation of 500 ms. The 20 most intense peptide ions with charge states ≥2 were isolated at a target value of 1,000 and fragmented by low-energy collision-induced dissociation (CID) with normalized collision energy of 30 and activation *Q* of 0.25. Dynamic exclusion settings of two repeat counts over 30 s and exclusion duration of 70 s.

### Database searching and protein identification

Peak lists were generated using extract-msn as part of Bioworks 3.3.1 (Thermo Scientific) using the following parameters: minimum mass 300, maximum mass 5,000; grouping tolerance 0.01 Da; intermediate scans 200; minimum group count 1; 10 peaks minimum and total ion current of 100. Peak lists for each LC-MS/MS run were merged into a single mascot generic format. Automatic charge state recognition was used because of the high-resolution survey scan (30,000). LC-MS/MS spectra were searched against the NCBI RefSeq database in a target decoy manner using MASCOT (v2.4, Matrix Science, UK). Search parameters used were as follows: fixed modification (carboamidomethylation of cysteine; +57 Da), variable modifications (oxidation of methionine; +16 Da), three missed tryptic cleavages, 20 p.p.m. peptide mass tolerance and 0.6 Da fragment ion mass tolerance. Peptide identifications with mascot ion score greater than the identity score were deemed significant. With a cut-off of two unique peptides per protein, <0.5% false discovery rate was achieved.

### Label-free spectral counting

The relative protein abundance between the samples was obtained by estimating the ratio of normalised spectral counts (RSc)^42^.


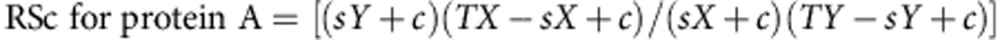


where *s* is the significant MS/MS spectra for protein A, *T* is the total number of significant MS/MS spectra in the sample, *c* is the correction factor set to 1.25, and *X* and *Y* are the whole apoptotic and apoptotic body-enriched samples, respectively. When RSc is <1, the negative inverse RSc value was used. Functional enrichment analysis was performed using FunRich software ( http://www.funrich.org)^43^.

### Immunoblotting

Samples were lysed in cytobuster protein extraction reagent (Novagen, Germany), analysed by SDS–PAGE and immunoblotted using the following dilutions: rabbit anti-pro caspase 3 (sc-7148, 1:2,000 dilution, Santa Cruz), mouse anti-β actin (A2228, clone AV-15, 1:4,000 dilution, Sigma-Aldrich), rabbit anti-histone H3 (9,715, 1:2,000 dilution, Cell Signalling, MA), rabbit anti-HMGB1 (3,935, 1:1,000 dilution, Cell Signalling), horseradish peroxidase-conjugated donkey anti-rabbit Ig (NA934, 1:10,000 dilution, GE Healthcare, UK) and horseradish peroxidase-conjugated sheep anti-mouse Ig (NA931, 1:4,000 dilution, GE Healthcare). Full scans of immunoblots are shown in Supplementary Fig. 13.

### Drug screening

Jurkat T cells and CFSE-stained^38^,^44^ Jurkat T cells stably expressing the PANX1 DN mutant were resuspend at a ratio of 1:1 in DMEM+0.5% BSA (1 × 10^6^ cells per ml). The cell mixture was induced to undergo apoptosis by anti-Fas treatment (250 ng ml^−1^) in the presence of 10 μM of compounds in the LOPAC^1280^ (Sigma-Aldrich) for 4 h at 37 °C, 5% CO_2_. Cells were then stained with TO-PRO-3 (0.67 μM) for 10 min at room temperature and immediately incubated on ice before analysis on a BD FACSCanto flow cytometer. FlowJo Software (Tree Star) was used to analyse the resultant flow cytometry data. Anti-Fas treatment for 4 h induced >90% of both cell types to undergo apoptosis. Necrotic cells were removed from analysis based on TO-PRO-3^high^ staining^12^. Apoptotic particles generated from Jurkat T cells and Jurkat T cells stably expressing the PANX1 DN mutant were distinguished based on the level of CFSE fluorescence. Apoptotic cells and apoptotic bodies were distinguished based on forward scatter (indicative of particle size)^12^.

### Statistical analyses

Unless otherwise described, the data are presented as means±s.e.m. To determine statistical significance between two groups (control versus a specific treatment), unpaired Student's two-tailed *t*-test was applied. A *P*-value of less than 0.05 was considered statistically significant. *P<0.05, **P<0.01, ***P<0.001.

## Additional information

**Accession codes:** Proteomic data sets are deposited in Vesiclepedia^19^ (accession number: Vesiclepedia_558)

**How to cite this article:** Atkin-Smith, G. K. *et al.* A novel mechanism of generating extracellular vesicles during apoptosis via a beads-on-a-string membrane structure. *Nat. Commun.* 6:7439 doi: 10.1038/ncomms8439 (2015).

## Supplementary Material

Supplementary Information Supplementary Figures 1-13.

Supplementary Dataset 1 A list of proteins identified in the proteomic analysis.
