# Supplementary material for: A novel mechanism of generating extracellular vesicles during apoptosis via a beads-on-a-string membrane structure
Source: Nat Commun. 2015 Jun 15;6:7439. doi: 10.1038/ncomms8439 (PMC4490561; doi:10.1038/ncomms8439)
Supplement: Supplementary Information — Supplementary Figures 1-13. [file ncomms8439-s1.pdf]

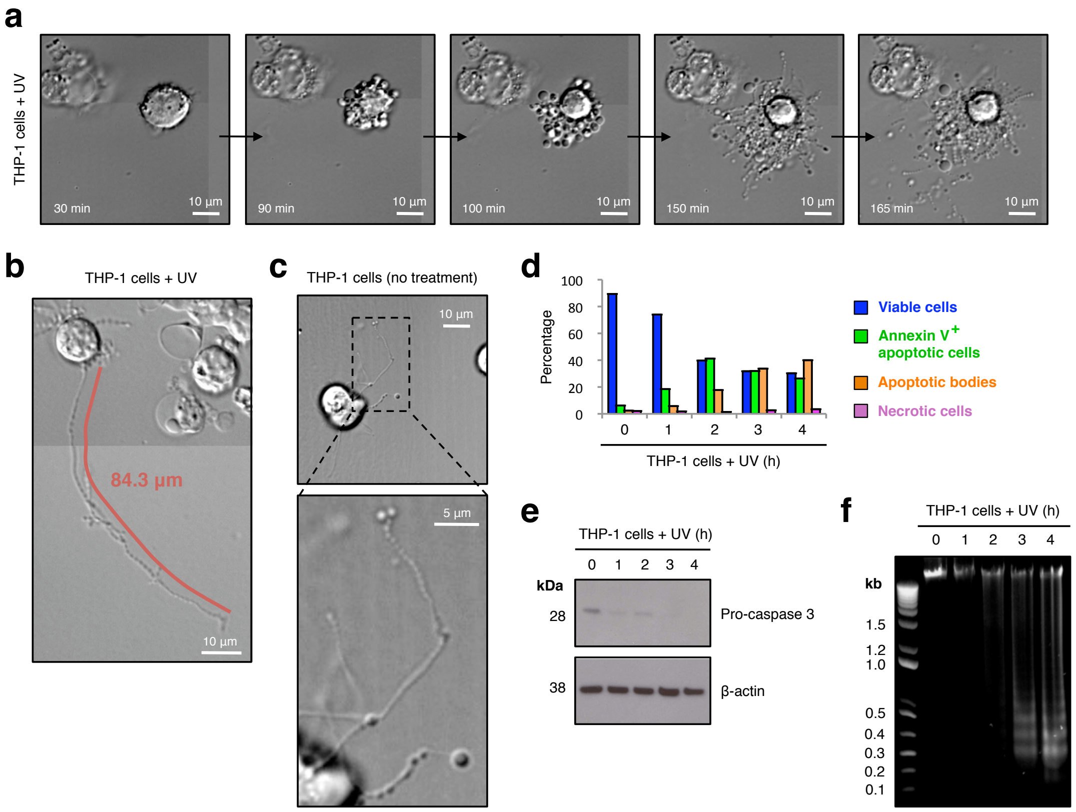


**Figure 1. Formation of beaded-apoptopodia by apoptotic human monocytes under various conditions. (a)** Time-lapse images monitoring THP-1 monocytes undergoing apoptosis. **(b)** Apoptotic THP-1 cells forming a strand of beaded-apoptopodia extending over 80 µm. **(c)** Formation of beaded-apoptopodia by THP-1 cells undergoing apoptosis spontaneously. **(d)** Apoptosis was induced and THP-1 cells were incubated for 1-4 h. The relative levels of viable cells, annexin $V^+$ apoptotic cells, apoptotic bodies and necrotic cells were monitored by flow cytometry and gated according to Supplementary Figure 3 ($n$ = 3). UV treatment promotes processing of pro-caspase-3 **(e)** and DNA fragmentation **(f)** in THP-1 cells. **a**,**b**,**d**,**e**,**f**, Apoptosis was induced by UV irradiation at 150 mJ $cm^{-2}$. Error bars in **d** represent s.e.m. Data are representative of at least two independent experiments.

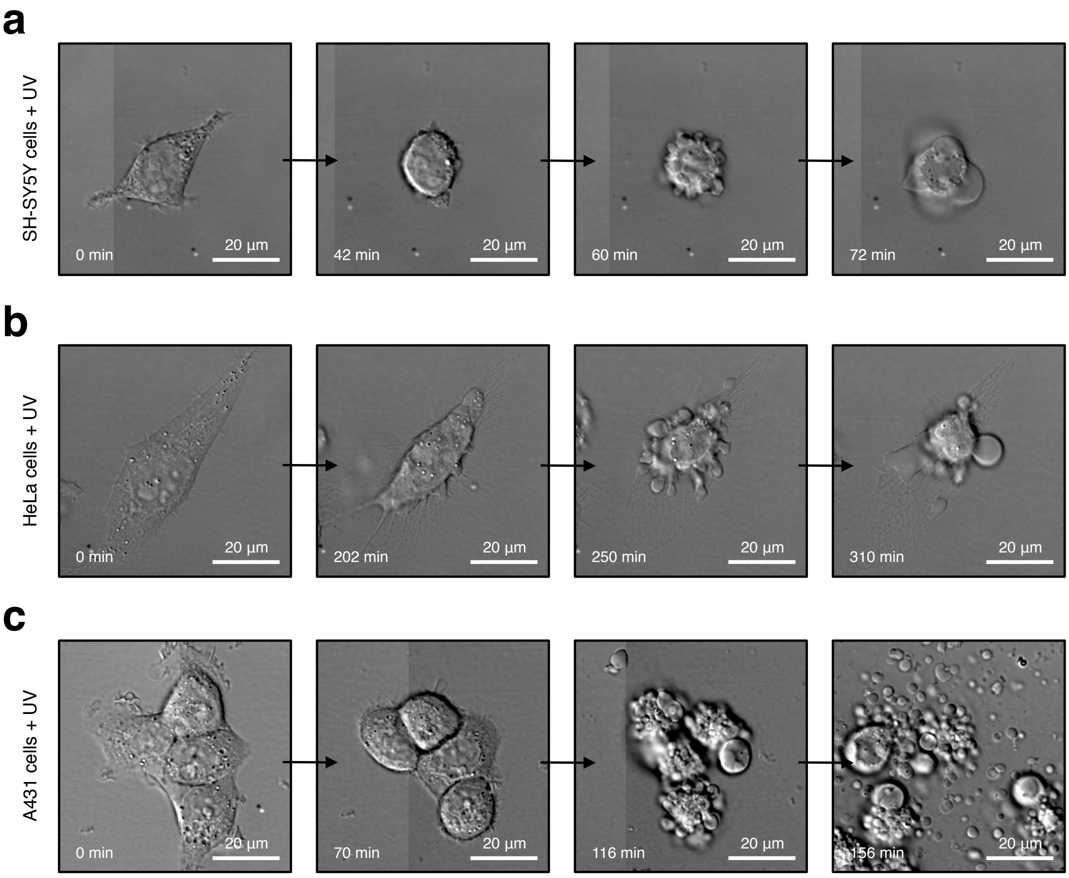


**Supplementary Figure 2. Neuronal cells, cervical epithelial cells and squamous epithelial cells do not generate beaded-apoptopodia during apoptosis.** Time-lapse images monitoring SH-SY5Y neuronal cells **(a)**, HeLa cervical epithelial cells **(b)**, and A431 squamous epithelial cells **(c)** undergoing UV-induced apoptosis. Data are representative of at least two independent experiments.

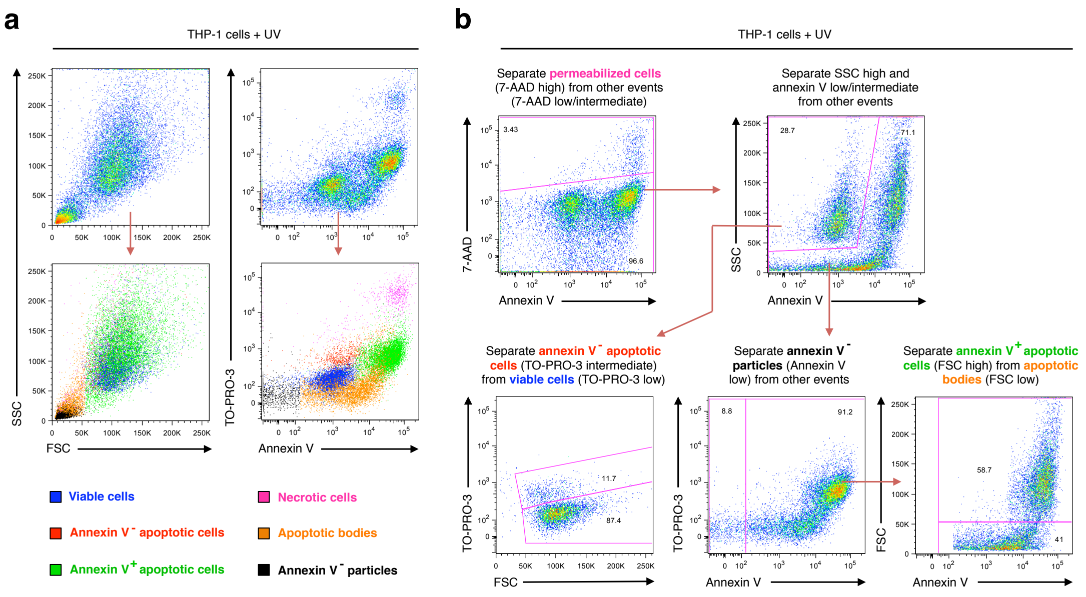


**Supplementary Figure 3. Electronic gating strategy for the separation of different cellular and subcellular population of THP-1 cells undergoing apoptosis *in vitro*. (a)** Flow cytometry analysis showing each type of cells and apoptotic particles gated according to **b**. **(b)** Flow cytometry analysis showing electronic gating strategy used to distinguish different types of dying/dead cells and subcellular vesicles.

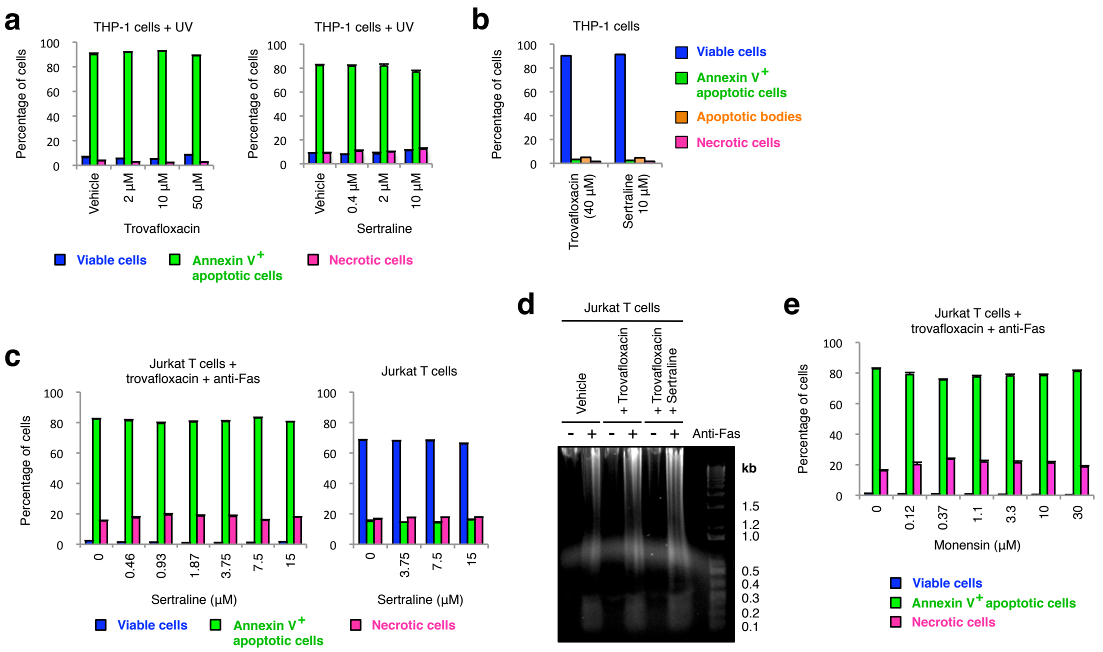


**Supplementary Figure 4. Trovafloxacin, sertraline and monensin do not affect the level of apoptosis. (a)** Trovafloxacin and sertraline do not interfere with THP-1 cell apoptosis induced by UV irradiation ($n$ = 3). **(b)** Trovafloxacin or sertraline alone do not induce THP-1 cells to undergo apoptosis or necrosis ($n$ = 3). **(c)** Sertraline does not interfere with Jurkat cell apoptosis induced by anti-Fas or induce Jurkat cells to undergo apoptosis or necrosis ($n$ = 3). **(d)** Trovafloxacin alone or trovafloxacin in combination with sertraline does not interfere with the fragmentation of DNA during apoptosis. **(e)** Monensin does not interfere with Jurkat cell apoptosis induced by anti-Fas ($n$ = 3). Error bars represent s.e.m. Data are representative of at least two independent experiments.

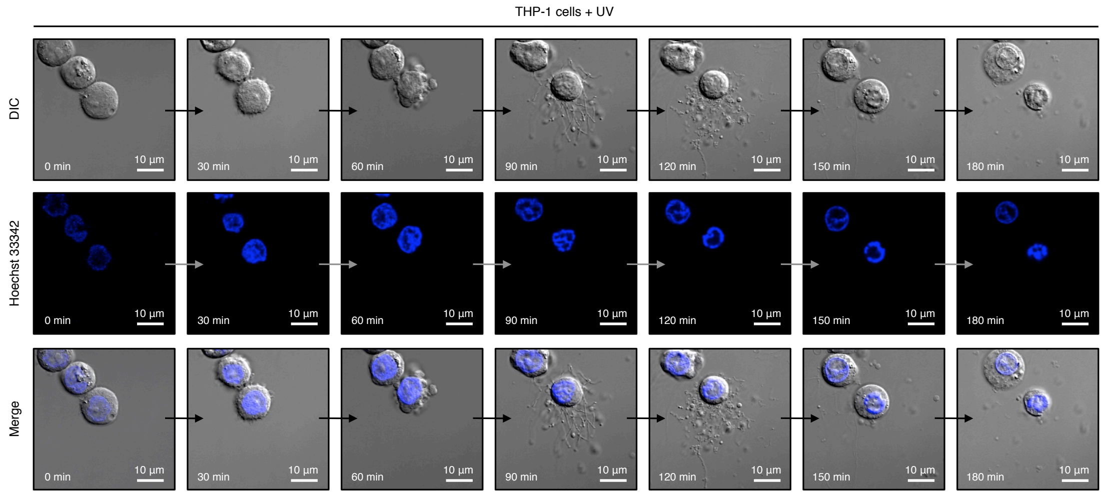


**Supplementary Figure 5. Localisation of nuclear DNA in THP-1 monocytic cells undergoing apoptosis.** Time-lapse images monitoring the morphology and localisation of nuclear DNA (based on Hoechst 33342 staining) in THP-1 cells undergoing UV-induced apoptosis. Data are representative of two independent experiments.

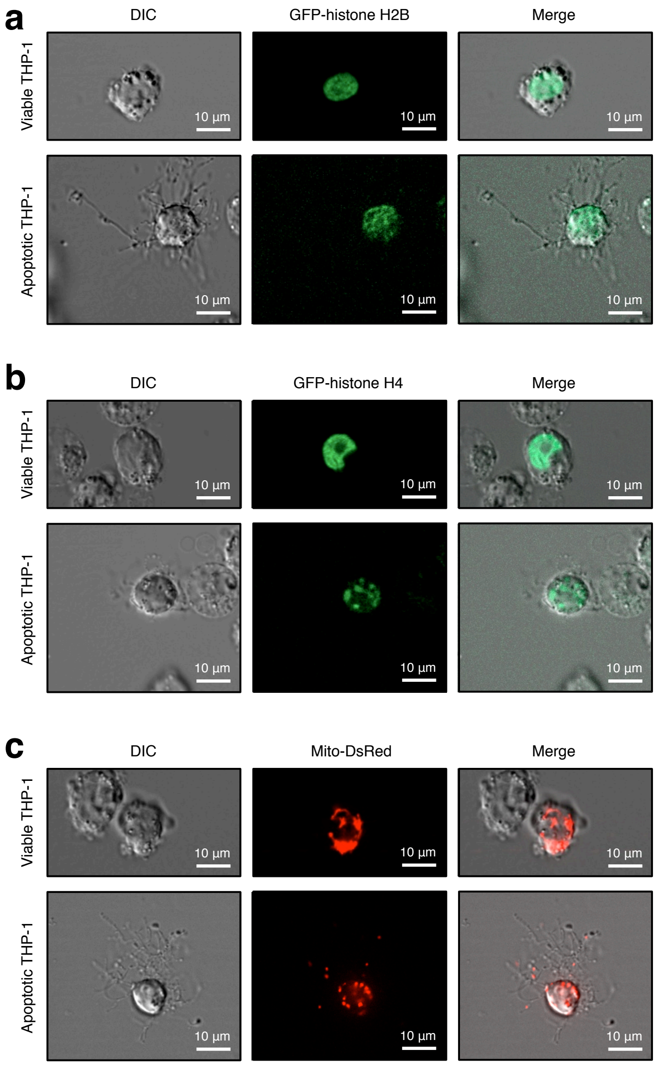


**Supplementary Figure 6. Localisation of nuclear and mitochondria proteins in viable and apoptotic THP-1 cells.** Morphology of viable and apoptotic THP-1 cells expressing GFP-histone H2B **(a)**, GFP-histone H4 **(b)** and Mito-DsRed **(c)** were monitored by confocal microscopy.

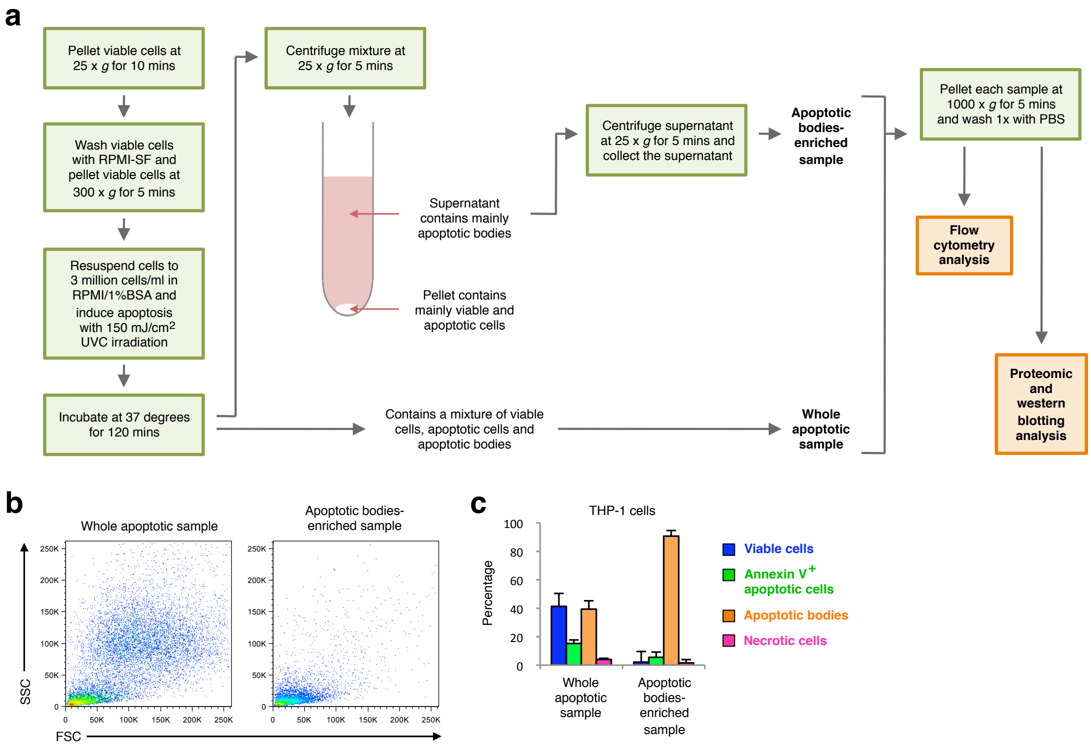


**Supplementary Figure 7. Enrichment of apoptotic bodies for flow cytometry, proteomic and western blotting analysis. (a)** Apoptotic bodies generated from apoptotic THP-1 cells were enriched by differential centrifugation as detailed in the schematic diagram. Apoptotic bodies-enriched sample and whole apoptotic sample (collected prior to differential centrifugation; containing a mixture of viable cells, apoptotic cells and apoptotic bodies) were subjected to flow cytometry, proteomic and western blotting analysis. **(b)** Flow cytometry analysis showing the size (FSC) and complexity (SSC) of particles in the whole apoptotic sample and apoptotic bodies-enriched sample. **(c)** The relative levels of viable cells, annexin $V^+$ apoptotic cells, apoptotic bodies and necrotic cells in the whole apoptotic sample and apoptotic bodies-enriched sample were monitored by flow cytometry (gated according to Supplementary Figure 3) ($n$ = 3). Error bars in **c** represent s.e.m. Data are representative of at least three independent experiments.

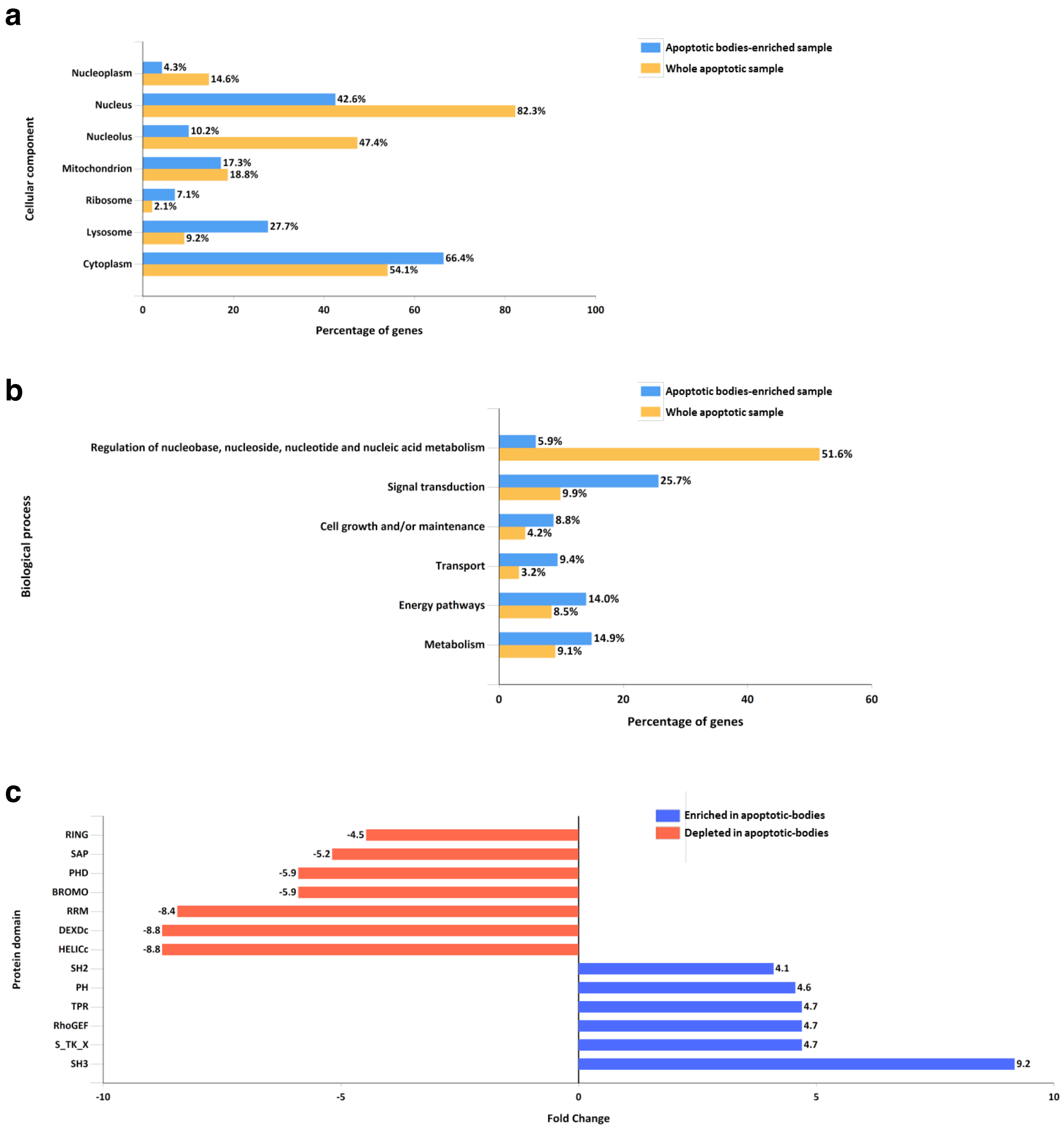


**Supplementary Figure 8. Functional enrichment analysis of proteins identified in apoptotic bodies-enriched sample compared to whole apoptotic sample.** Cellular components **(a)** and biological processes **(b)** overrepresented in highly abundant proteins in apoptotic bodies-enriched sample and whole apoptotic sample. **(c)** Protein domains that are enriched or depleted in apoptotic bodies-enriched sample compared to whole apoptotic sample. Data in **a-c** are depicted based on FunRich analysis software.

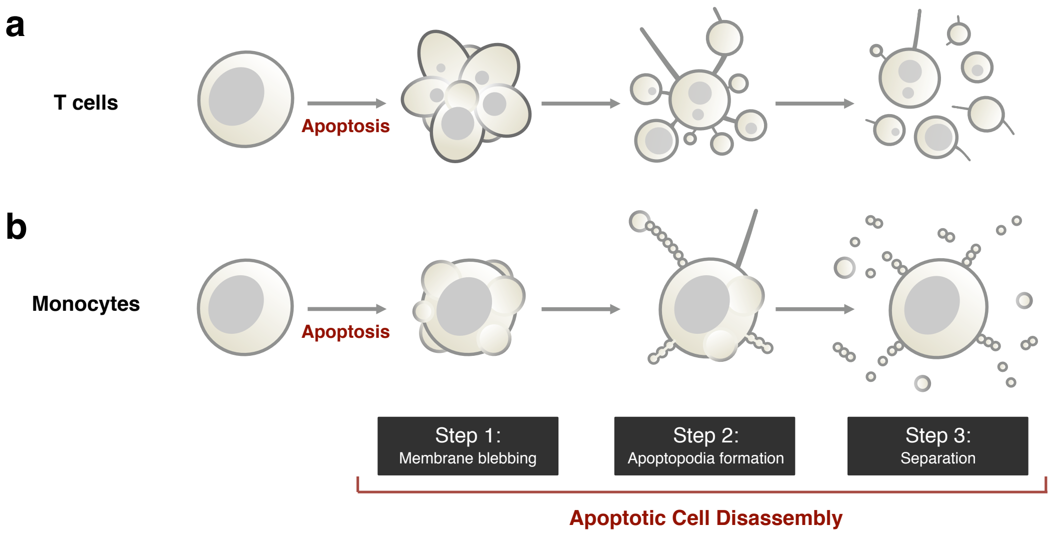


**Supplementary Figure 9. Schematic of T cells and monocytes undergoing apoptotic cell disassembly.** **(a)** Apoptotic T cells first undergo dynamic membrane blebbing, and subsequent formation of a thin string-like membrane protrusion (known as apoptopodia) facilitates the parting of membrane blebs to generate apoptotic bodies. Finally, separation of apoptotic bodies occurs when the connecting apoptopodia is broken off (possibly via shear force). **(b)** For apoptotic monocytes, small membrane blebs are formed at early stages of apoptosis. At later stages of apoptosis when membrane blebbing is beginning to cease, thin membrane protrusions are generated at the periphery of the apoptotic cell. These membrane protrusions can become segmented to form a 'beads-on-a-string' membrane structure (denote as beaded-apoptopodia). Fragmentation of beaded-apoptopodia can subsequently generate membrane-bound vesicles characterised as apoptotic bodies. It is worth noting that the differences in the dynamics of membrane blebbing between T cells (e.g. Jurkat T cells) and monocytes (e.g. THP-1 cells) can potentially be due to differences in membrane composition and activities of actomyosin contraction regulators (e.g. ROCK1, PAK2 and LIMK1).

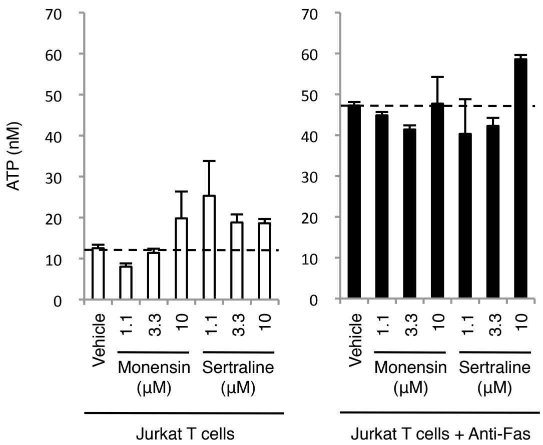


**Supplementary Figure 10. Sertraline and monensin does not have a major effect on the release of ATP during apoptosis.** Jurkat cells were treated with sertraline and monensin in the absence or presence of apoptotic stimulus (anti-Fas) for 4 h. The level of ATP in the cultured supernatant was monitored by a luciferase/luciferin-based assay ($n$ = 3). Error bars represent s.e.m. Data are representative of two independent experiments.

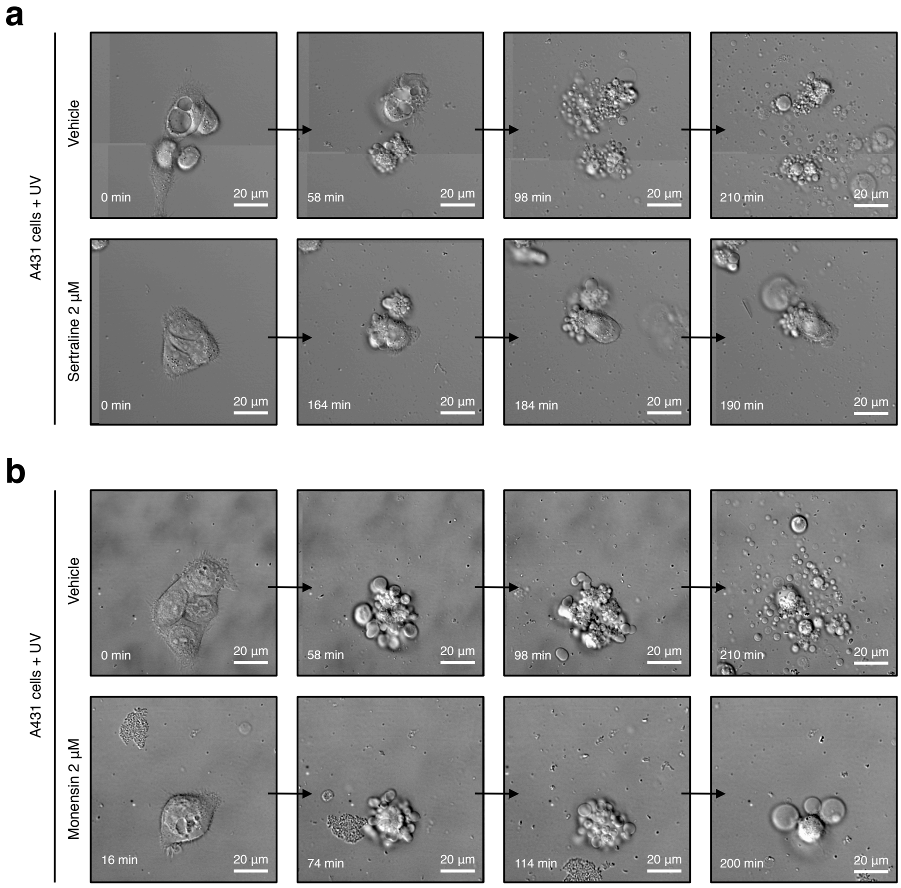


**Supplementary Figure 11. Sertraline and monensin inhibit the formation of apoptotic bodies from squamous epithelial (A431) cells.** Time-lapse images monitoring the disassembly of A431 squamous epithelial cells treated with or without sertraline **(a)** or monensin **(b)**. Data are representative of at least two independent experiments.

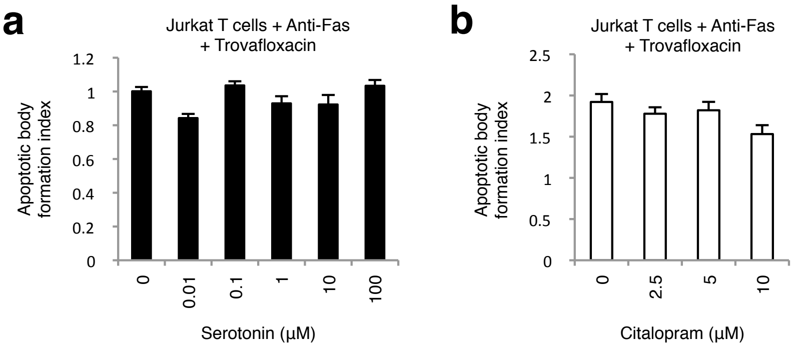


**Supplementary Figure 12. Inhibition of serotonin reuptake is unlikely the mechanism underpinning the effect of sertraline on apoptotic cell disassembly. (a)** Increasing the extracellular concentration of serotonin does not inhibit the formation of apoptotic bodies from Jurkat cells under conditions when PANX1 channels are blocked ($n$ = 3). **(b)** Citalopram (a selective serotonin reuptake inhibitor) does not block apoptotic body formation by Jurkat cells under conditions when PANX1 channels are inhibited ($n$ = 3). Error bars represent s.e.m. Data are representative of at least three independent experiments.

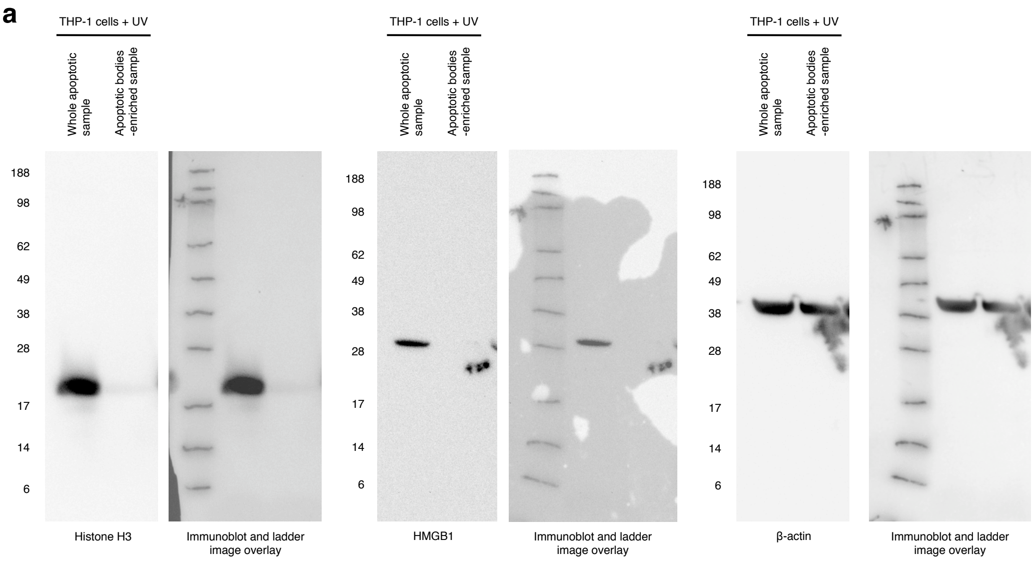


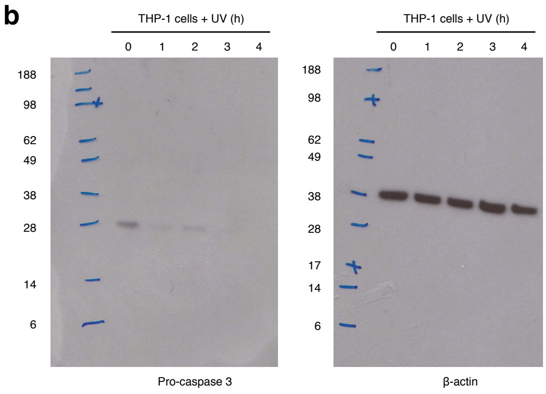


**Supplementary Figure 13. Full scans of immunoblots.** Full scans of immunoblots corresponding to Figure 2f **(a)** and Supplementary Figure 1e **(b)**.
